# Accidental Intrathecal Administration of Digoxin in an Elderly Male with End-Stage Renal Disease

**DOI:** 10.1155/2017/9072018

**Published:** 2017-08-22

**Authors:** Claudia Martin, Kitae Kevin Park, Antonio Liu

**Affiliations:** ^1^Department of Family Medicine, White Memorial Medical Center, Los Angeles, CA, USA; ^2^Department of Internal Medicine, White Memorial Medical Center, Los Angeles, CA, USA; ^3^Department of Neurology, Loma Linda School of Medicine, Loma Linda, CA, USA

## Abstract

The systemic effects of digoxin toxicity have been well-known. However, there has been no case citing the effects of intrathecal digoxin in light of end-stage renal disease in the elderly. Here, we report on the case of the successful management of accidental intrathecal digoxin administration in an elderly male with end-stage renal disease.

## 1. Introduction

Digoxin is a cardiac glycoside used as an inotrope in heart failure [1]. The intrathecal administration of digoxin has been cited in the past [2]. Its effects range in severity and duration. However, limited data exists on the effects and management of intrathecal digoxin administration. Here we report on the case of a 52-year-old old male with multiple comorbidities who underwent accidental intrathecal digoxin administration during an elective surgery and its successful management.

## 2. Case Description

This is 52-year-old male with a complicated medical history significant for end-stage renal disease on hemodialysis three times a week, admitted for elective total hip hemiarthroplasty. Spinal anesthesia was attempted twice and it was realized that approximately 0.4 mg of digoxin had been accidentally injected into the intrathecal space. The hemiarthroplasty was converted to general endotracheal anesthesia and completed. While being in the recovery room, patient began complaining of symmetric, bilateral lower extremity weakness with subsequent paraplegia and upper extremity “heaviness,” with consequent sensory deficits to the C4-C5 levels. Patient was taken immediately for MRI of cervical, thoracic, and lumbar spine and brain CT, which were negative for spinal hematoma and cord compression. One dose of Methylprednisolone 2500 mg IV was given and patient placed on a Methylprednisolone drip at 5.4 mg/hr for the following 24 hours. Six hours after initiation of drip patient became agitated and confused. At this time, labs remarkable for potassium level 6.4 mEq/L (5.6 mEq/L prior to surgery, baseline 5.2 mEq/L) and digoxin 1.9 ng/ml. ECG showed no acute changes pertaining to digoxin toxicity or hyperkalemia. Patient was given Calcium Gluconate and Regular Insulin with 1 amp of D50W. Urgent hemodialysis was performed and 1.0 L of fluid was removed. Patient began complaining incessantly of dyspnea: ABG: 7.35/42/83/23.2 on 2 L nasal cannula, SpO_2_ 100%. Vital signs were temperature 97.5 F, blood pressure 171/80, pulse 76 beats per minute, and respirations 18. Troponin was negative. Concurrently, patient was also observed to be posturing with a right-sided gaze. Repeat brain CT showed no acute changes. EEG showed no seizure activity. After, patient was noted to be less anxious and breathing comfortably, though not oriented to time, person, or place. Digoxin level noted to be at 1.4 ng/mL. By postoperative day 2, patient was alert and oriented to person and place. On postoperative day 3, patient had 2.5 L of fluid removed through scheduled hemodialysis. Digoxin level was 1.1 ng/mL. By the evening, patient was able to engage in full conversation, was moving all four extremities, and was following simple commands.

## 3. Discussion

Digoxin toxicity contributed to an estimated 5,000 emergency department (ED) visits per year from 2005 to 2010 [3, 4]. Such ED visits were a record of adverse drug events as defined by serum digoxin concentration and laboratory parameters. The latter contrasts to events described in this case report which delineates the management of accidental intrathecal digoxin administration in an elderly male with end-stage renal disease.

Digoxin is a reversible inhibitor of the sodium/potassium-ATPase pump found in every cell in the body. The sodium/potassium-ATPase pump acts as a receptor for digoxin [5]. Specifically, in the myocardium, the latter effect of digoxin leads to increased heart contractility, following the elevation of intracellular sodium concentration in exchange for intracellular potassium. Digoxin is excreted for the most part by the kidneys, with 70–82% excreted unchanged in urine, and in the normal kidney has a half-life of 50 hours as opposed to 3–5 days in anuric patients [3, 6, 7]. However, the extent to which such data is applicable to intrathecal digoxin administration is questionable as the latter pertains to systemic circulation. In this manner, expectant management based on clinical improvement, in collaboration with the primary team, intensivists, and specialists, served to successfully treat the accidental intrathecal administration of digoxin in an elderly male with end-stage renal disease.

Inhibition of the sodium/potassium-ATPase pump in neurons may explain the paralysis observed in the patient presented in this case report. Different isoforms of this receptor (sodium/potassium-ATPase pump) produce different sensitivities and effects in the human body. To illustrate, the alpha-2 isoform is found in glia as well as myocardium; however, neurons have the alpha-3 isoform which has a higher affinity to digoxin. In contrast, nephrons, as well as other tissues, have the alpha-1 isoform which is characterized by lower affinity to digoxin [2, 5]. The latter may explain the sequence of cell function inhibition by digoxin administration, with the first cells affected being neurons, followed by glia, myocytes, and then all other cells in the body.

In an animal study involving intrathecal digoxin in rabbits, motor weakness and sensory deficits were observed in less than 10 minutes within administration [2]. Similarly, literature also describes the case of a 21-year-old healthy male who complained of paresthesia and paraplegia after 2 hours of accidental intrathecal digoxin, with complete resolution of motor, sensory, and spinal reflex function after 24 hours [1]. Although digoxin immune Fab therapy may function similarly in a patient with decreased renal function, neither digoxin nor Fab can be removed by hemodialysis which was believed that it would complicate the removal of toxins and anesthesia further in this case [8]. Though other interventions were considered, this report exemplifies a case illustrating the successful expectant medical management of accidental intrathecal digoxin in an elderly male with end-stage renal disease with full resolution of neurological deficits.

## Abbreviations

CT: Computed tomography
D50W: Dextrose 50% in water
Digoxin Immune Fab: Digoxin immune antigen binding fragments binding fragments (Fab)
ECG: Electrocardiogram
EEG: Electroencephalogram
ED: Emergency department
MRI: Magnetic resonance imaging.
